# Polyphenol and Tryptophan Contents of Purple Corn (*Zea mays* L.) Variety KND and Butterfly Pea (*Clitoria ternatea*) Aqueous Extracts: Insights into Phytochemical Profiles with Antioxidant Activities and PCA Analysis

**DOI:** 10.3390/plants12030603

**Published:** 2023-01-30

**Authors:** Juthamat Ratha, Chawalit Yongram, Panyada Panyatip, Patcharapol Powijitkul, Pimolwan Siriparu, Suthida Datham, Aroonsri Priprem, Tarapong Srisongkram, Ploenthip Puthongking

**Affiliations:** 1 Melatonin Research Group, Faculty of Pharmaceutical Sciences, Khon Kaen University, Khon Kaen 40002, Thailand; 2 Division of Cannabis Health Science, College of Allied Health Sciences, Suansunandha Rajabhat University, Samut Songkhram 75000, Thailand; 3 Department of Pharmacognosy, Faculty of Pharmacy, Srinakharinwirot University, Nakhon Nayok 26120, Thailand; 4 Faculty of Pharmacy, Mahasarakham University, Maha Sarakham 44150, Thailand; 5 Division of Pharmaceutical Chemistry, Faculty of Pharmaceutical Sciences, Khon Kaen University, Khon Kaen 40002, Thailand

**Keywords:** purple corn, butterfly pea, aqueous extract, anthocyanins, phenolics, melatonin, PCA

## Abstract

Plants are a rich source of phytochemical compounds with antioxidant activity. Several studies have revealed that the consumption of plant polyphenols reduces the risk of diseases. Purple corn (*Zea mays* L. variety KND) and butterfly pea (*Clitoria ternatea*; CT) were selected to be investigated as alternative natural polyphenol sources to increase the value of these plants. Phytochemical profiles and antioxidant activities of KND cob, silk, husk and CT extracts alone and in combination were investigated in this study. The results revealed that purple corn cob (C) extract had the highest tryptophan, melatonin, total anthocyanin (TAC) and delphinidin content, while the purple corn silk (S) extract showed the highest total phenolic content (TPC) and antioxidant activities. Serotonin was found only in purple corn husk (H) extract and C extract. High contents of tryptophan and sinapic acid were found in CT extract. Principal component analysis (PCA) revealed that strong antioxidant activities were strongly correlated with protocatechuic acid and *p*-hydroxybenzoic acid contents, moderate antioxidant activities were strongly correlated with melatonin, and low antioxidant activities were strongly correlated with sinapic acid content. Therefore, the purple corn variety KND waste cobs, silk and husks are a potentially rich source of health-promoting phytochemical compounds.

## 1. Introduction

Polyphenols, such as anthocyanins, are phytochemicals [1] that have been reported to be health-promoting compounds as they can decrease the damaging oxidative stress associated with many diseases [2,3]. Moreover, the indolamine derivatives of the essential amino acid tryptophan, serotonin and melatonin are widely used as dietary supplements to regulate mood and sleep [4,5]. There are many reports that support the antioxidant activity and safety of plant polyphenols [2,6,7] and the consumption of plant polyphenols has been reported to reduce the risk of inflammatory diseases, diabetes, aging, neurodegenerative diseases, cancers and cardiovascular diseases [8,9].

Cultivated purple corn (*Zea mays* L.) contains a variety of phytochemical constituents, such as anthocyanins, tryptophan, melatonin, and other phenolic compounds [10]. Purple corn extracts have been reported to have many biological activities, including antioxidant, anti-inflammatory, anticarcinogenic, and antimicrobial activities [11,12]. Phytochemical compounds accumulate in all parts of purple corn, and anthocyanin contents were found to be high in the cob, husk, and silk [12,13]. To date, there have been no reports of the quantitative analysis of phenolics, melatonin and its metabolite contents in the different parts of the Thai purple corn variety KND (Khao Niew Dum) [12,14,15].

Butterfly pea (*Clitoria ternatea*; CT) is a flower that produces anthocyanins in its petals. In Thailand, CT has been widely used for a variety of purposes. For example, CT is commonly used as a natural colorant in many beverages, foods, and cosmetics in South–East Asia. In the medical community, CT is used in Ayurvedic medicine for health-promoting applications [16,17,18]. In particular, Ayurvedic medicine use the leaves and roots of CT to treat neurological disorders [17]. Vankar and Srivastava [19] stated that CT flowers showed the highest anthocyanin content and antioxidant activity among a survey of 15 red and blue flowers. Anthocyanins from CT have also exhibited anti-inflammatory, antioxidant, antimicrobial, and anticancer properties [20,21].

Examination and identification of the phytochemical contents of the different parts of purple corn variety KND and CT in Thailand could increase the value of these plants as alternative sources of existing phytochemicals or through the discovery of novel compounds with applications in the pharmaceutical industry and in health promotion. For example, one health benefit from phytochemical compounds in plant might the reduction in the oxidative stress by their acting as antioxidants [9]. The objectives of this study were, therefore, to analyze the contents of phytochemical compounds, including phenolic acids, tryptophan, melatonin, and anthocyanins, in the different parts of purple corn variety KND and CT, as well as the combined extracts of both plants. Antioxidant capacities were analyzed to elucidate the relationship between phytochemical compounds and antioxidant capacity using principal component analysis (PCA) and classification analysis.

## 2. Results and Discussion

### 2.1. Phytochemical Profiles

Plants are a rich source of polyphenols (phenolic acids, flavonoids, anthocyanins) and nitrogenous compounds, which have many biological functions [1,22]. In this study, phytochemical profiles including the contents of phenolic acids, anthocyanin, tryptophan, serotonin, and melatonin were investigated in anthocyanin plants extracts.

**Figure 1:**
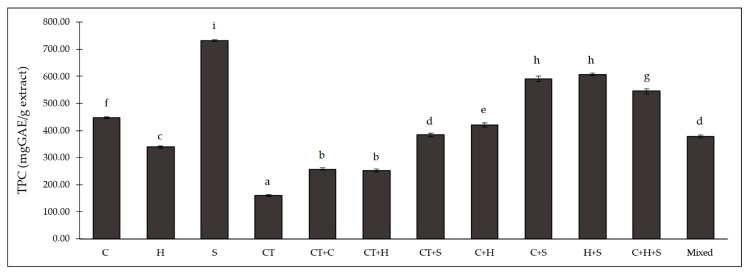
Total phenolic acid content (TPC) in different extracts from purple corn and butterfly pea. C: cob extract; H: husk extract; S: silk extract; CT: butterfly pea extract; CT + C: butterfly pea and cob extract; CT + H: butterfly pea and husk extract; CT + S: butterfly pea and silk extract; C + H: cob and husk extract; C + S: cob and silk extract; H + S: husk and silk extract; C + H + S: cob, husk, and silk extract; mixed: combination of butterfly pea, cob, husk, and silk extracts. The different superscript letters (a–i) indicate a significant difference between each content of the extracts using Tukey’s test (*n* = 3, *p* < 0.05).

Total phenolic content (TPC) among all of the extracts varied from 160 to 732 mg GAE/g extract (Figure 1). The highest TPC was in the purple corn silk (S) extract, which concurs with the results of Mohamed et al. [10], who also found higher TPC in the silk than in other parts of corn. Moreover, the TPC ranges for all of the purple corn extracts were within those seen in other purple corn studies (range 1.5 to 1400 mg GAE/g extract) [6,13,23]. The TPC of the CT extract (160 mg GAE/g extract) was higher than the range measured by Jeyaraj et al. (41 to 65 mg GAE/g extract) [2]. Differences might be due to the accumulation of polyphenols in different parts of plants, which are related to the function of those compounds in the plant’s lifecycle and the growth phase [24,25]. The combination H + S (606.56 mg GAE/g extract) and C + S (589.93 mg GAE/g extract) extracts showed the highest TPC, followed by C + H + S, C + H, CT + S (383 mg GAE/g extract) and mixed (376 mg GAE/g extract) extracts (Figure 1). According to the study of Priprem et al. [26], an anthocyanin complex (AC) containing purple corn cob and butterfly pea (32 mg GAE/g extract) extracts had a TPC about eight-times lower than the CT + C extract (257 mg GAE/g extract). These results revealed that the combined extracts not only reduced requirements for time and raw materials, but also enhanced the TPC in combined extracts, particularly the combinations with purple corn silk (S).

According to the quantitative analysis using HPLC, the phenolic compounds present in the extracts were composed of gallic acid (GA), protocatechuic acid (PCCA), *p*-hydroxybenzoic acid (*p*-HO), chlorogenic acid (ChA), vanillic acid (VA), *p*-coumaric acid (*p*-CA), ferulic acid (FA), and sinapic acid (SA), all of which are primarily found in plants. The relative phenolic acid abundance was in the range of 2.90–11.17 mg/g extract, as shown in Table 1. For individual extracts, the S extract showed the highest relative abundance and contained PCCA, *p*-HO, VA, and GA. The main phenolic acids presented in the C extract were PCCA, *p*-CA, VA, and *p*-HO, whereas the phenolics in the CT extract were SA and GA. ChA and *p*-CA, as well as FA, were also detected in the C and H extracts. In particular, PCCA, *p*-HO and VA were the major phenolic acids observed in all the purple corn extracts, while SA was the main phenolic acid in CT extract. This result confirms a previous report of Kapcum et al. [13], who found PCCA to be the major phenolic compound in the pericarp, silk, cob, and tassel, parts of purple corn, whereas *p*-HO was found at concentrations of 0.09 mg/g in silk and 0.20 mg/g in cob. Previously, purple corn has been reported to contain major phenolic acids, such as GA, PCCA, *p*-HO, ChA, VA, *p*-CA, FA, and SA [13,27], while CT contains other substances, such as kaempferol, quercetin, rutin, and epicatechin [2,18,28]. For the combined extracts (Table 1), the highest relative phenolic abundance was found in the H + S extract, followed by the C + S and C + H + S extracts. These combined extracts presented a significantly higher level of relative abundance than that found in the S extract, H extract and C extract, respectively. Interestingly, the CT + S extract (5.45 mg/g extract) showed a 1.3-fold increase in the total phenolic acid contents compared with the content of CT extract (4.23 mg/g extract), whereas 6.08 mg/g extract was found for the mixed extract. In addition, the combination of each part of KND showed a synergistic effect that could increase the value of KND agricultural waste. Moreover, this combination of plants has been reported to prevent disease; in particular, an anthocyanin complex (AC) prepared from the mixing of purple corn (*Z. mays*) cob and CT extracts was previously shown to have anti-inflammatory activity and promote collagen production [29].

**Figure 2:**
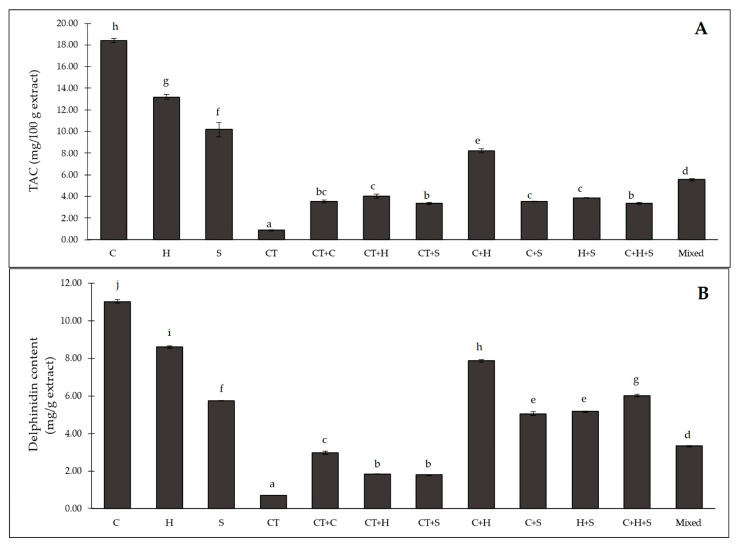
(**A**) Total anthocyanin content (TAC) and (**B**) delphinidin content in different extracts from purple corn and butterfly pea. C: cob extract; H: husk extract; S: silk extract; CT: butterfly pea extract; CT + C: butterfly pea and cob extract; CT + H: butterfly pea and husk extract; CT + S: butterfly pea and silk extract; C + H: cob and husk extract; C + S: cob and silk extract; H + S: husk and silk extract; C + H + S: cob, husk, and silk extract; mixed: combination of butterfly pea, cob, husk, and silk extracts. The different superscript letters (a–j) indicate a significant difference between each content of the extracts using Tukey’s test (*n* = 3, *p* < 0.05).

The total anthocyanin content (TAC) values ranged between 0.86 and 18.39 mg/100 g extract. The greatest TAC value was observed in the C extract (18.39 mg/100 g extract), followed by the H (13.20 mg/100 g extract) and S (10.18 mg/100 g extract) extracts (Figure 2A). The TAC results obtained from all of the aqueous KND extracts in this study were higher than those reported for ethanolic extracts of purple corn in a previous study [7,30]. For the combined extracts, the C + H extract (8.20 mg/100 g extract) showed the highest TAC, followed by the mixed extract (5.54 mg/100 g extract), the combination of CT + H (4.01 mg/100 g extract) and the combination of H + S (3.85 mg/100 g extract), as shown in Figure 2A. The CT + C extract showed a lower TAC than the anthocyanin complex (AC) from Priprem et al. [26], which could be due to the adding of an anthocyanin co-pigment for AC formation.

Delphinidin is one of the major anthocyanin substances responsible for the deep blue-to-purple color in plants and is a candidate for health promotion in pharmaceutical usage [31]. The delphinidin content results corresponded with the TAC results with values in the range of 0.69–11.02 mg/g extract (Figure 2B). The C extract exhibited a significantly higher delphinidin content than the other individual extracts, while the CT extract showed the lowest delphinidin content. The C + H (7.86 mg/g extract) showed a high delphinidin content, followed by C + H + S (6.02 mg/g extract), C + S (5.05 mg/g extract), and H + S (5.16 mg/g extract). For the CT combined extract, mixed (CT + C + H +S ) provided 3.33 mg/g extract, followed by CT + C extract (2.96 mg/g extract). The CT + S extract (1.79 mg/100 g extract) was not significantly different from the CT + H extract (1.82 mg/100 g extract), although the lowest delphinidin content was found in the CT extract (0.69 mg/100 g extract), as presented in Figure 2B. These results suggest that the cob and husk part of purple corn are a rich resource of anthocyanins, as we reduced each raw material by half in the extraction step. Thus, both C and H, which are by-products of harvest, could potentially be excellent sources of health-promoting bioactive compounds.

The higher TPC and anthocyanin contents obtained in this study compared to other studies could due to the high polarity of water used for extraction and the capability of polyphenol dissolved in water from its numerous hydroxy groups. The amino acid tryptophan is a precursor for serotonin and melatonin via the shikimate pathway. As shown in Table 2, the tryptophan content in individual extracts was highest in the C extract, followed by CT, (26.74 µg/g extract) and H extract (24.82 µg/g extract). The S extract showed the lowest tryptophan level. For the indolamine metabolites, the content of serotonin and melatonin was high in the C and H extracts; however, they were not detected in the S and CT extracts. This study showed that all parts of KND contained tryptophan, which has previously been identified in other varieties of corn [32] and in butterfly pea [28]. The level of melatonin was correlated with the level of tryptophan. Our results also showed a remarkably higher melatonin content than in other corns, which have been reported in the range of 1.37 to 93.63 ng/g extract [15,32,33].

In a previous publication, Priprem et al. [26] investigated the combination of purple corn cob and butterfly pea extracts in an anthocyanin complex and found that this mixture showed potential anti-inflammatory and collagen promotion effects in a human forehead fibroblast cell line. This study is different from the previous study because the different parts of purple corn and butterfly pea were mixed without the formation of the anthocyanin complex. The phytochemical constituents and antioxidant capacities of the extract alone and in combination were determined in the current study. We highlight that the highest contents for anthocyanin, tryptophan and its metabolites were in the C extract. Alternative tryptophan sources were from the CT and H extracts as they showed tryptophan content values that were statistically insignificant. The best source for phenolic compounds and strong antioxidant capacity was the S extract. These observations suggest that purple corn variety KND and CT are potential alternative agricultural sources of bioactive supplements and can be further promoted as a food supplement or a good source of value-added raw material in the pharmaceutical industry.

### 2.2. Antioxidant Capacities

The antioxidant capacities of the individual extracts were evaluated using three different approaches: ABTS, DPPH and FRAP assays. In the ABTS assay, the IC_50_ values of the extracts ranged from 8 to 75 µg/mL, as presented in Table 3. The highest and lowest capacities were found in the S and CT extracts, respectively. The current study revealed a higher ABTS capacity from various parts of the corn (*Zea mays* L.) extract via ABTS assay than a previous study (range from 219 to 800 µg/mL) [23]. In the DPPH assay, the highest antioxidant activity was found in the S extract, and the activity among extracts ranged from 17 to 207 µg/mL. Chaiyasut et al. [34] reported that the IC_50_ value of aqueous CT extract was 130 µg/mL and 135 µg/mL in ABTS and DPPH assays, respectively, which are lower antioxidant capacities than the ones presented in this study. The CT extract has higher antioxidant capacity because it contained more anthocyanins than the extracts tested in the study of Chaiyasut [34] and the anthocyanins act as antioxidants by donating a hydrogen ion. The range of FRAP values among the extracts was from 3.36 (in CT extract) to 17.48 (in S extract) mmole/g extract. Interestingly, significantly strong antioxidant capacities were observed in the S extract, with capacities about 2- to 2.5-fold lower than Trolox. The combination of plants part extracts significantly enhanced their antioxidant capacity. Higher antioxidant activities (ABTS, DPPH and FRAP assays) were observed in the combination of C + S, followed by H + S and C + H + S (Table 3). The ABTS, DPPH and FRAP assays are widely used to evaluate antioxidant capacity in plant extracts and can measure antioxidant activity in hydrophilic systems [35]. Using the antioxidant capacities of the individual materials, it can be seen that the strongest combined antioxidant activity would come from any combination with the silk extract. Although hydroxycinnamic acid has a stronger antioxidant capacity than hydroxybenzoic acid [36], the *p*-CA and FA, which are members of hydroxycinnamic acid, were found at low concentrations in cob and husk. In contrast, PCCA, *p*-HO and VA, as the major hydroxybenzoic acid in silk, provide a strong antioxidant capacity. According to Kapcum et al. [13] and Dong et al. [6], corn silk extract has a higher antioxidant potential than extracts from the other parts of corn. The high content of anthocyanins and phenolics might improve antioxidant capacity. Our findings correlate with the results of Khamphasan et al. [12], who postulated that high anthocyanin contents might result in high total phenolic compounds contents, which positively correlate with their antioxidant capacities.

### 2.3. Pearson’s Correlation Coefficients Analysis

Pearson’s correlation coefficient (*r*) values between phytochemical composition and antioxidant capacity of purple corn and butterfly pea extracts are presented in Table 4. Strong and significant correlation between TPC and antioxidant capacity, including the ABTS (*r* = 0.739), DPPH (*r* = 0.960) and FRAP (*r* = 0.823) assays, were obtained. Both PCCA and *p*-HO showed a positive and significant correlation with either TPC or antioxidant capacity (*r* > 0.5). VA was positively correlated with antioxidant capacity (ABTS; *r* = 0.370, DPPH; *r* = 0.457 and FRAP; *r* = 0.560). On the other hand, a negative correlation was seen in all antioxidant capacities against SA (ABTS; *r* = −0.492, DPPH; *r* = −0.703 and FRAP; *r* = −0.699). Low correlations were observed between antioxidant capacities and GA, ChA, *p*-CA, and FA contents (−0.191 < *r* < 0.244). Interestingly, the major phenolic acids (PCCA, *p*-HO, and VA) found in purple corn were correlated with antioxidant activity. Our finding supports a previous study that revealed that phenolic acids were related to antioxidant activity [2,6,7,10,30]. Delphinidin (*r* = 0.627) and TAC (*r* = 0.613) were moderately correlated with FRAP values. Indolamine compounds (Try, 5-HT, and Trp) showed a weak correlation with antioxidant capacity (−0.150 < *r* < 0.438).

### 2.4. Classification Analysis Based on the Principal Component Analysis

**Figure 3:**
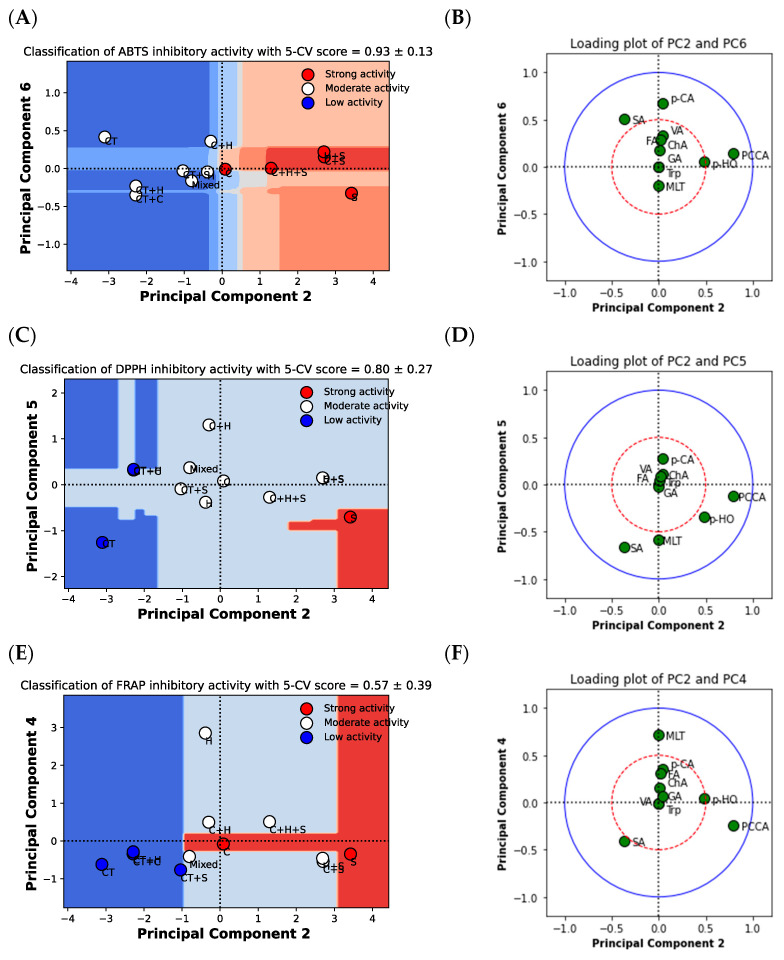
Principal component analysis, decision boundary and loading plots between the phytochemical components and antioxidant inhibitory activities of 12 samples. (**A**) Score plot with decision boundary between PC2 and PC6. The samples were classified based on the strong (red circle) and moderate (white circle) ABTS inhibitory activities. (**B**) Loading plot between PC2 and PC6. The red and blue lines determine the correlation of phytochemical components between PC2 and PC6 at 0.5 and 1.0, respectively. (**C**) Score plot with decision boundary between PC2 and PC5. The samples were classified based on the strong (red circle), moderate (white circle), and low (blue circle) DPPH inhibitory activities. (**D**) Loading plot between PC2 and PC5. The red and blue lines determine the correlation of phytochemical components between PC2 and PC5 at 0.5 and 1.0, respectively. (**E**) Score plot with decision boundary between PC2 and PC4. The samples were classified based on the strong (red circle), moderate (white circle), and low (blue circle) FRAP inhibitory activities. (**F**) Loading plot between PC2 and PC4. The red and blue lines determine the correlation of phytochemical components between PC2 and PC4 at 0.5 and 1.0, respectively.

Figure 3 shows the classification analysis based on PCA and the loading plot between antioxidant activities (i.e., strong, moderate and low activity of each ABTS, DPPH and FRAP inhibitory effect) and the phytochemical components of the 12 extracts. Figure 3A shows the ABTS inhibitory activity, which includes strong (i.e., C, S, C + S, H + S, and C + H + S) and moderate activity (i.e., H, CT, CT + C, CT + H, CT + S, C + H, and mixed), and is best classified by PC2 and PC6 with a five-fold CV accuracy score of 0.93 ± 0.13. Figure 3B shows the loading plot of PC2 and PC6 using a correlation circle, which is weakly correlated at 0.5 (red) and strongly correlated at 1.0 (blue). The result shows that protocatechuic acid was strongly correlated (*r* > 0.5) with the strong ABTS inhibitory activity, while the sinapic acid was correlated (*r* > 0.5) with the moderate ABTS inhibitory activity.

Figure 3C shows that the DPPH inhibitory activity was categorized into three classes, namely strong (i.e., S), moderate (i.e., C, H, CT + C, CT + S, C + H, C + S, H + S, C + H + S, and mixed) and low inhibitory activity (i.e., CT, and CT + H) by PC2 and PC5, with a five-fold CV accuracy score of 0.80 ± 0.27. Figure 3D shows that strong and moderate DPPH inhibitory activities were strongly correlated with protocatechuic acid and *p*-hydroxybenzoic acid (*r* > 0.5), while moderate activity was strongly correlated with melatonin (*r* > 0.5), and low activity was strongly correlated with sinapic acid (*r* > 0.5).

Figure 3E shows that FRAP inhibitory activity was categorized into three classes, namely strong (i.e., S, and C), moderate (i.e., H, C + H, C + S, H + S, C + H + S, and mixed) and low inhibitory activity (i.e., CT, CT + C, CT + S, and CT + H) by PC2 and PC4 with a five-fold CV accuracy score of 0.57 ± 0.39. Figure 3F shows that strong and moderate FRAP inhibitory activity were strongly correlated with protocatechuic acid. The moderate FRAP inhibitory activity was also strongly correlated with *p*-hydroxybenzoic acid (*r* > 0.5) and melatonin (*r* > 0.5), while the weak FRAP inhibitory activity was moderately correlated with sinapic acid.

As previously mentioned, the main phenolics found in purple corn, including *p*-HO and PCCA, were correlated with strong antioxidant activities. The results also show that the strong antioxidant activities were in accordance with high overall phenolic acid content. Melatonin exhibited a correlation with moderate antioxidant activities, while SA was correlated with low antioxidant activity. Pandi and Kalappan [37] summarized that the antioxidant properties of SA included anti-diabetic, anti-inflammatory, and cardioprotective effects. Melatonin is well-known as an antioxidant based on its amphiphilic molecule, small size, solubility in water and lipids, and ability to penetrate all components of our cells [38]. The major polyphenols were correlated with the strong and moderate antioxidant activities found in purple corn variety KND waste.

This combination of plants has been previously reported to have the potential to prevent disease. In particular, the anthocyanin complex from the mixing of purple corn (*Z. mays*) cob and CT showed anti-inflammatory activity and promoted collagen production [29]. Moreover, purple corn waxy (*Z. mays* L.) and ginger extracts have been reported to show synergistic effects on the prevention of retinopathy in diabetic rats [39].

## 3. Materials and Methods

### 3.1. Chemicals

Folin–Ciocalteu reagent was purchased from Merck (Darmstadt, Germany), and potassium persulfate, ferric chloride, ferrous sulfate, di-sodium hydrogen phosphate, and phosphoric acid from Univar (Ajax Finechem, Taren Point, Australia). 1,1-diphenyl-2-picrylhydrazyl (DPPH) was obtained from Fluka Chemikal (Buchs, Switzerland). 2,2′-azino-bis-3-ethylbenzthiazoline-6-sulphonic acid (ABTS), 2,4,6-tripyridyl-S-triazine (TPTZ), and 6-hydroxy-2,5,7,8-tetramethyl chromane 2-carboxylic acid (Trolox) were obtained from Sigma Aldrich (St. Louis, MO, USA). Standard chemicals, including tryptophan, serotonin, delphinidin, gallic acid (GA), protocatechuic acid (PCCA), *p*-hydroxybenzoic acid (*p*-HO), chlorogenic acid (ChA), vanillic acid (VA), *p*-coumaric acid (*p*-CA), ferulic acid (FA) and sinapic acid (SA), were products of Sigma Aldrich (St. Louis, MO, USA). Standard melatonin was obtained from Shanghai Chemical (Hubei, China). Trifluoroacetic acid and acetonitrile were supplied by ACI Labscan (Bangkok, Thailand). All organic solvents and reagents for HPLC were of an analytical grade.

### 3.2. Materials and Sample Extraction

**Figure 4:**
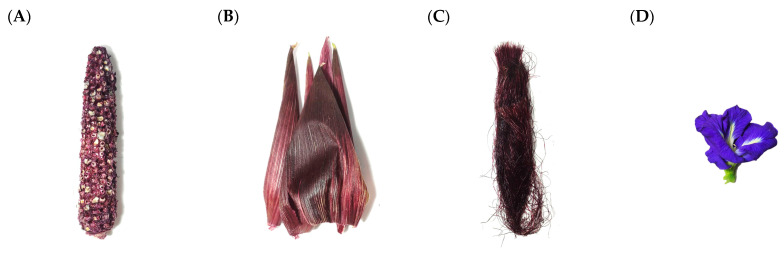
Raw materials: (**A**) KND cob, (**B**) KND husk, (**C**) KND silk and (**D**) butterfly pea.

Fresh cobs (C), husks (H) and silks (S) of purple corn (*Zea mays* L. var *ceratina* Kulesh; genotype KKU-WX111031) variety KND were acquired from the Plant Breeding Research Center for Sustainable Agriculture, Faculty of Agriculture, Khon Kaen University, Khon Kaen, Thailand. Butterfly pea (*Clitoria ternatea*; CT) was purchased at a local market in Khon Kaen, Thailand, in 2021 (Figure 4). All materials were dried, cut into small pieces and ground using a blender (model BL2A0166, Tefal, Shaoxing, China).

In brief, the dried ground sample (6.50 g) was extracted using distilled water (390 mL) at 90 °C for 30 min as in Table 5. The amount of samples was fixed at 6.50 g, and the ratio of sample to distilled water was 1:20, following the method of Priprem et al. [29]. The extract was filtrated using a vacuum filtration and the residual was subjected to re-extraction with the same conditions. The extraction was conducted in triplicate. The filtrates were combined and lyophilized using a freeze-dryer.

### 3.3. Determination of Total Anthocyanin Content (TAC)

The total anthocyanin content (TAC) was analyzed using the pH-differential method according to Yang and Zhai [40]. In brief, the dilution sample was placed into a 10 mL volumetric flask and made up to a final volume with pH 1.0 buffer and the other with pH 4.5 buffer. Absorbance was measured using a UV–visible spectrophotometer (Genesys^TM^ 180, Thermo scientific, Waltham, MA, USA) at 520 and 700 nm, respectively. The experiment was performed in triplicate (*n* = 3). TAC was expressed as milligrams of cyaniding-3-glucoside equivalent per g of dry weight (mg/100 g extract), and the value was calculated as in Equation (1):

(1) $$TAC(mg/100\;g\;\mathrm{extract})=\frac{AB}{\varepsilon L}\times Mw\times D\times \frac{V}{G}\times 1000$$

where AB is the absorbance of the sample (A_520nm_-A_700nm_)_pH1.0_–(A_520nm_-A_700nm_)_pH4.5_; Mw is the molecular weight of cyanidin-3-glucoside (449.2 g/mol); D is the dilution factor of the sample; V is the final volume of the sample (mL); ε is the molar coefficient of cyanidin-3-glucoside (26,900 L/mol/cm); L is the cuvette path length (1 cm); and G is the weight of the sample (g).

### 3.4. Total Phenolic Content (TPC)

The total phenolic content (TPC) was determined using Folin–Ciocalteu reagent based on the procedure of Song et al. [41] with a minor modification. In brief, 50 µL of each crude extract (400 µg/mL) was mixed with 50 µL Folin–Ciocalteu reagent (10%), and the mixture was incubated for 8 min in darkness. Afterwards, 100 µL of 7.5% sodium carbonate was added and incubated for 30 min in darkness. The absorbance was measured at a wavelength of 760 nm. The experiment was performed in triplicate (*n* = 3). TPC was determined using gallic acid as standard (0.5–20 µg/mL) and was calculated with a calibration curve of gallic acid (y = 0.0457x + 0.058; R^2^ = 0.9991). The results were reported as mg of gallic acid equivalent (GAE) per gram of dry weight.

### 3.5. High Performance Liquid Chromatography (HPLC) Analysis

#### 3.5.1. Identification and Quantification of the Phenolic Compounds by HPLC

The extracts were filtered with a 0.45 μm filter membrane before subsequent analysis using HPLC (Agilent 1200 series) and using a Hichrom5 C18 column of 4.6 × 250 mm with a diode array detector. The detection wavelength was set at 280 nm for hydroxybenzoic acids, gallic acid (GA), protocatechuic acid (PCCA), *p*-hydroxybenzoic acid (*p*-HO), and vanillic acid (VA), and at 320 nm for hydroxycinnamic acids, chlorogenic acid (ChA), *p*-coumaric acid (*p*-CA), ferulic acid (FA) and sinapic acid (SA). The gradient elution was carried out using 1% (*v*/*v*) acetic acid in water (mobile phase A) and acetonitrile (mobile phase B), as described by Siriparu et al. [42]. The system was as follows: 0–5 min, 5% mobile phase B; 5–15 min, 5–9% mobile phase B; 15–22 min, 9–11% mobile phase B; 22–38 min, 11–18% mobile phase B; 38–43 min, 18–23% mobile phase B; 43–44 min, 23–90% mobile phase B; 44–55 min, 90–80% mobile phase B; 55–65 min, 80–5% mobile phase B; and 65–70 min, 5% mobile phase B for re-equilibration. The flow rate was set at 0.8 mL/min, the column temperature was set at 40 °C, and the injection volume was set at 20 μL. The experiment was performed in triplicate (*n* = 3). Phenolics were identified as equivalents of the aforementioned standards. The analytical method showed LOD and LOQ of phenolic acids as 0.001 µg/mL and 0.01 µg/mL, as described from our previous report [42].

#### 3.5.2. Delphinidin Analyzed by HPLC

High-performance liquid chromatography (Agilent 1260 series) was used to separate delphinidin into 12 samples according to Goufo et al. [43]. Standard delphinidin was dissolved in methanol. The delphinidin concentration was prepared at 10, 15, 30, 50, 80 and 200 µg/mL. The crude extracts were dissolved with distilled water at 10 mg/mL. The sample separation was performed using a Hichrom5 C18 column (4.6 × 250 mm) at 520 nm. A gradient system used 0.1% trifluoroacetic acid (TFA) in distilled water and 0.1% TFA in acetonitrile as mobile phases A and B, respectively. The separation was carried out for 65 min with a 20 µL injection volume at a flow rate of 1.0 mL/min. The gradient program used the following profiles: 0% B in 0–5 min, 0–20% B in 5–15 min, 20–50% B in 15–30 min, 50–100% B in 30–45 min, 100% B in 45–50 min and 100–0% B in 50–55 min and 0% B in 55–65 min. The experiment was performed in triplicate (*n* = 3). The equation of the standard curve was y = 55.818x − 655.96; R^2^ = 0.9989 (y is the peak area and x is delphinidin concentration). The delphinidin contents were expressed in mg/g extract.

#### 3.5.3. Tryptophan Serotonin and Melatonin Analyzed by HPLC-FD

The KND cob (C), husk (H), silk (S) and butterfly pea (CT) extracts were transferred to a Strata^®^ C18-E solid phase extraction (SPE) cartridge (Phenomenex, Torrance, CA, USA). Methanol was passed through SPE by gravity flow. Afterward, the filtrate was collected for further analysis. The serotonin, tryptophan and melatonin were identified using high-performance liquid chromatography (Agilent 1260, Santa Clara, CA, USA) equipped with an Unisol C18 column (5µm, 4.6 × 250 mm), following the method of Siriparu et al. [42]. The wavelength of the fluorescence detector (FD) was set at 286 nm for excitation and 346 nm for emission. Mobile phase A was methanol, while mobile phase B was 50 mM phosphate buffer (pH 4.5). The experiment was performed in triplicate (*n* = 3). The analytical method was validated according to ICH guideline. The values of the limit of detection (LOD) and limit of quantification (LOQ) were 0.0025 µg/mL and 0.05 µg/mL, as described from our previous report [42].

### 3.6. Determination of Antioxidant Capacity

#### 3.6.1. ABTS Assay

2,2′-Azino-bis-3-ethylbenzthiazoline-6-sulphonic acid (ABTS) assay was performed according to the method of Kim et al. [44]. A sample aliquot of 0.1 mL was mixed with 0.1 mL ABTS reagent. The ABTS solution consisted of a mixture of 7 mM ABTS and 140 mM potassium persulfate. The reagent was incubated for 12–16 h in the dark. The solution was then diluted with distilled water in a 1:15 *v*/*v* ratio. The mixture was mixed and retained at room temperature in the dark for 30 min. The absorbance was measured at 734 nm, and the experiment was performed in triplicate (*n* = 3). The ABTS activity was expressed as the percentage inhibition using Equation (2):

(2) $$\;\mathrm{ABTS}\;\mathrm{inhibition}\;\left(\%\right)=\frac{\left(\;\mathrm{Absorbance}\;\mathrm{of}\;\mathrm{control}-\mathrm{Absorbance}\;\mathrm{of}\;\mathrm{sample}\right)\times 100}{\;\mathrm{Absorbance}\;\mathrm{of}\;\mathrm{control}}$$

Trolox was used as a reference standard. The ABTS activity was expressed as 50% of the inhibitory concentration (IC_50_) value by plotting of ABTS inhibition versus extract concentrations.

#### 3.6.2. DPPH Assay

1,1-Diphenyl-2-picrylhydrazyl (DPPH) assay was adapted from Arabshahi-Delouee and Urooj [45]. An aliquot (0.1 mL) of each sample was added to 0.1 mL DPPH solution (200 µM)and the reaction mixture was incubated for 30 min in the dark. Absorbance was measured at 517 nm. The experiment was performed in triplicate (*n* = 3). DPPH inhibition was calculated using Equation (3):

(3) $$\mathrm{DPPH}\;\mathrm{inhibition}\;\left(\%\right)=\frac{\left(\mathrm{Absorbance}\;\mathrm{of}\;\mathrm{control}-\mathrm{Absorbance}\;\mathrm{of}\;\mathrm{sample}\right)\times 100}{\;\mathrm{Absorbance}\;\mathrm{of}\;\mathrm{control}\;}$$

The DPPH capacity was reported in the term of the half maximal of inhibitory concentration (IC_50_) value. The IC_50_ values were constructed by plotting the percentage inhibition against the concentration of the extract. The IC_50_ values were computed as the same ABTS assay.

#### 3.6.3. FRAP Assay

Ferric reducing antioxidant power (FRAP) assay was modified from the method of Iqbal et al. [46]. A total of 20 µL of the extracts was mixed with 80 µL FRAP solution, and retained for 5 min, and the absorbance was measured at 595 nm. The FRAP solution was freshly prepared by mixing acetate buffer (pH 3.6), 10 mM TPTZ, and 20 mM ferric chloride in a 10:1:1 *v*/*v* ratio. Before being used, the solution was warmed at 37 °C for 30 min. Ferrous sulfated (0–200 µM) was used to generate the standard curve and the experiment was performed in triplicate (*n* = 3). The standard curve was obtained (y = 0.0058x + 0.0855; R^2^ = 0.9988). FRAP values were expressed as mM Fe^2+^/g extract.

### 3.7. Classification Analysis

Classification analyses were performed to determine the relationship between phytochemical components (i.e., tryptophan, melatonin, gallic acid, protocatechuic acid, *p*-hydroxybenzoic acid, chlorogenic acid, vanillic acid, *p*-coumaric acid, ferulic acid, and sinapic acid) and the antioxidant activities (i.e., ABTS, DPPH, and FRAP inhibitory activities). The extracts were labeled based on their bioactivity classes (i.e., strong activity, moderate activity, and low activity). For ABTS and DPPH inhibitory activities, the bioactivity classes were classified based on the IC_50_ against ABTS, and DPPH, which are IC_50_ < 20 µg/mL (strong activity), IC_50_ < 100 µg/mL (moderate activity), and IC_50_ ≥ 100 µg/mL (low activity). For FRAP inhibitory activity, the bioactivity classes were classified based on FRAP values, including FRAP values ≥ 1000 (strong activity), 500 < FRAP values ≤ 1000 (moderate activity), and FRAP values ≤ 500 (low activity). Then, the PCA with data labels were first classified using the k-nearest neighbors (kNN), Naive Bayes (NB), support vector machine (SVM), reinforcement learning (RF), decision tree (DT), and gradient boosting classification (XGBoost) algorithms. The algorithms with the highest five-fold cross validation (CV) accuracy score were selected to build the second classification. Moreover, the top two principal components (PCs) from the selected algorithms were used to describe the contribution of phytochemicals components to the antioxidant activities. The accuracy matrix was calculated as in Equation (4) based on the ratio of summation of the true positive (TP) and true negative (TN) divided by the summation of true positive (TP), true negative (TN), false positive (FP) and false negative (FN).

Accuracy = TP + TN/(TP + TN + FP + FN)(4)

The score plots between two PCs with decision boundaries and the loading features were plotted to visualize the Pearson’s correlation of phytochemicals components and the antioxidant activities. The decision boundaries are the predicted classes from classification analyses, where random forest (RF) was used for ABTS and DPPH inhibitory activity classification, and decision tree (DT) was used for FRAP inhibitory activity classification.

### 3.8. Statistical Analysis

All the experiments were carried out in triplicate, and all the data are reported as means ± standard deviation (SD). One-way analysis of variance (ANOVA) and Tukey comparison test (*p* < 0.05) were employed using SPSS software (SPSS Inc., Chicago, IL, USA). Classification analysis was performed using scikit-learn and Python 3.9 software.

## 4. Conclusions

This study shows that the phytochemical composition of aqueous extracts of cob, husk, and silk of purple corn variety KND includes tryptophan, melatonin, serotonin, delphinidin, TAC, TPC, and phenolic acids. Additionally, butterfly peas are a good source of tryptophan, sinapic acid and gallic acid. Interestingly, we found that purple corn cob has the highest tryptophan content, and purple corn silk presented a high antioxidant capacity. According to the evaluation of the combination of both raw materials, the presence of polyphenols and antioxidant activity significantly increased, especially in mixtures with purple corn silk extract to enhance phenolic acids, and the mixture of purple corn cob and husk. The current study also reveals a strong relation between phenolic acid content and antioxidant activity. Cob and husk could be rich potential alternative sources of tryptophan, delphinidin, and melatonin, and silk could be a potential source of anthocyanins and antioxidants. Both purple corn and butterfly pea are, therefore, valuable sources to increase multifunctional biological properties, as well as possible supplements in food or as raw materials in the pharmaceutical industry.

## Figures and Tables

**Table 1:** Phytochemical compositions analyzed using HPLC of difference extracts obtained from purple corn and butterfly pea.

Sample	Phenolics Contents								
	GA	PCCA	*p*-HO	ChA	VA	*p*-CA	FA	SA	Relative Phenolic Abundance
	(mg/g Extract)								
C	NQ	2.60 ± 0.01 ^e^	1.03 ± 0.06 ^c^	0.65 ± 0.00 ^b^	1.23 ± 0.01 ^e^	1.61 ± 0.00 ^f^	0.96 ± 0.00 ^d^	NQ	8.08 ± 0.06 ^f^
H	NQ	2.05 ± 0.01 ^c^	0.89 ± 0.01 ^b^	1.03 ± 0.01 ^e^	0.76 ± 0.01 ^c^	1.75 ± 0.00 ^h^	1.61 ± 0.01 ^f^	NQ	8.08 ± 0.02 ^f^
S	0.54 ± 0.01 ^d^	5.84 ± 0.06 ^g^	2.68 ± 0.03 ^e^	NQ	0.56 ± 0.02 ^b^	NQ	NQ	ND	9.62 ± 0.10 ^g^
CT	0.58 ± 0.00 ^f^	NQ	NQ	ND	NQ	NQ	NQ	3.65 ± 0.02 ^d^	4.23 ± 0.02 ^b^
CT + C	NQ	0.58 ± 0.01 ^a^	NQ	NQ	0.52 ± 0.01 ^a^	NQ	NQ	1.81 ± 0.02 ^b^	2.91 ± 0.01 ^a^
CT + H	0.57 ± 0.00 ^e^	0.59 ± 0.00 ^a^	NQ	NQ	NQ	0.63 ± 0.01 ^a^	0.57 ± 0.01 ^a^	1.84 ± 0.02 ^b^	4.20 ± 0.01 ^b^
CT + S	0.59 ± 0.00 ^g^	2.07 ± 0.01 ^c^	0.78 ± 0.00 ^a^	ND	NQ	NQ	NQ	2.01 ± 0.02 ^c^	5.45 ± 0.01 ^c^
C + H	NQ	2.17 ± 0.01 ^d^	0.85 ± 0.02 ^ab^	0.79 ± 0.01 ^d^	0.92 ± 0.01 ^d^	1.67 ± 0.01 ^g^	1.07 ± 0.02 ^e^	NQ	7.47 ± 0.04 ^e^
C + S	0.51 ± 0.00 ^c^	5.01 ± 0.02 ^f^	2.48 ± 0.03 ^d^	ND	0.87 ± 0.01 ^d^	0.91 ± 0.01 ^c^	0.68 ± 0.01 ^b^	NQ	10.46 ± 0.06 ^i^
H + S	0.49 ± 0.00 ^a^	4.95 ± 0.02 ^f^	2.55 ± 0.02 ^d^	0.68 ± 0.00 ^c^	0.79 ± 0.05 ^c^	0.99 ± 0.01 ^d^	0.73 ± 0.00 ^b^	NQ	11.17 ± 0.04 ^j^
C + H + S	NQ	1.71 ± 0.01 ^b^	5.01 ± 0.04 ^f^	0.61 ± 0.01 ^a^	0.81 ± 0.01 ^c^	1.12 ± 0.01 ^e^	0.83 ± 0.02 ^c^	NQ	10.10 ± 0.05 ^h^
Mixed	0.50 ± 0.00 ^b^	2.06 ± 0.01 ^c^	0.80 ± 0.04 ^a^	NQ	NQ	0.72 ± 0.01 ^b^	0.59 ± 0.00 ^a^	1.41 ± 0.02 ^a^	6.08 ± 0.03 ^d^

^a–j^ Different superscript letters within each column indicate significant differences (Tukey’s test, *n* = 3, *p* < 0.05). ND denotes not detected. NQ denotes not quantified (the value below 0.5 mg/g extract). GA: gallic acid (RT = 8.31; y = 3.6752x − 4.8683; R^2^ = 0.9999); PCCA: protocatechuic acid (RT = 13.53; y = 1.8027x − 0.3566; R^2^ = 0.9996); *p*-HO: *p*-hydroxybenzoic acid (RT = 22.75; y = 1.3627x + 5.5192; R^2^ = 0.9995); ChA: chlorogenic acid (RT = 24.69, y = 1.2981x − 0.5234; R^2^ =0.9991); VA: vanillic acid (RT = 29.07; y = 1.6616x + 2.7813; R^2^ = 0.9991); *p*-CA: *p*-coumaric acid (RT = 41.58; y = 5.1252x + 1.2145; R^2^ = 0.9999); FA: ferulic acid (RT = 46.19; y = 4.6847x − 1.036; R^2^ = 1.0000); SA: sinapic acid (RT = 46.92; y = 4.3675x − 1.3617; R^2^ = 1.0000). C: cob extract; H: husk extract; S: silk extract; CT: butterfly pea extract; CT + C: butterfly pea and cob extract; CT + H: butterfly pea and husk extract; CT + S: butterfly pea and silk extract; C + H: cob and husk extract; C + S: cob and silk extract; H + S: husk and silk extract; C + H + S: cob, husk, and silk extract; mixed: combination of butterfly pea, cob, husk, and silk extracts.

**Table 2:** The content of serotonin, tryptophan and melatonin in each individual extract analyzed using HPLC–FD.

Compound	Amount (µg/g Extract)			
	Purple Corn Cob (C) Extract	Purple Corn Husk (H) Extract	Purple Corn Silk (S) Extract	Butterfly Pea (CT) Extract
Serotonin	3.62 ± 0.27 ^b^	3.05 ± 0.06 ^a^	NQ	NQ
Tryptophan	621.89 ± 7.00 ^c^	24.82 ± 1.20 ^b^	11.58 ± 0.37 ^a^	26.74 ± 0.02 ^b^
Melatonin	7.85 ± 0.52 ^b^	3.25 ± 0.04 ^a^	NQ	NQ

^a–c^ Different superscript letters within each row indicate significant differences (Tukey’s test, *n* = 3, *p* < 0.05). NQ denotes not quantified (values of less than 1.0 µg/g extract). Serotonin (RT = 4.34; y = 3.2343x + 0.7243; R^2^ = 0.9973); tryptophan (RT = 7.75; y = 0.6764x + 0.0759; R^2^ = 0.9993); melatonin (RT = 29.83; y = 2.2283x + 0.4306; R^2^ = 0.9973).

**Table 3:** Antioxidant capacities of combined extracts obtained from purple corns and butterfly peas.

Sample	IC_50_ Value (µg/mL)		FRAP Assay (mmole/g Extract)
	ABTS Assay	DPPH Assay	
C	16.64 ± 0.30 ^e^	45.80 ± 1.59 ^f^	11.77 ± 0.20 ^c^
H	24.52 ± 0.12 ^h^	74.20 ± 0.30 ^i^	8.91 ± 0.15 ^d^
S	8.58 ± 0.05 ^b^	17.24 ± 0.43 ^b^	17.48 ± 0.30 ^b^
CT	74.60 ± 0.34 ^k^	207.68 ± 2.34 ^l^	3.36 ± 0.07 ^h^
CT + C	35.30 ± 0.10 ^i^	97.50 ± 0.77 ^j^	3.04 ± 0.04 ^hi^
CT + H	46.60 ± 0.19 ^j^	130.46 ± 0.93 ^k^	2.77 ± 0.02 ^i^
CT + S	20.67 ± 0.31 ^f^	71.16 ± 0.12 ^h^	4.91 ± 0.16 ^g^
C + H	20.79 ± 0.34 ^fg^	53.06 ± 0.06 ^g^	6.31 ± 0.23 ^f^
C + S	11.91 ± 0.06 ^c^	23.56 ± 0.25 ^c^	8.90 ± 0.11 ^d^
H + S	11.84 ± 0.36 ^c^	34.40 ± 0.21 ^d^	8.40 ± 0.22 ^e^
C + H + S	13.41 ± 0.09 ^d^	40.17 ± 1.48 ^e^	8.18 ± 0.15 ^e^
Mixed	21.43 ± 0.12 ^g^	75.93 ± 0.94 ^i^	5.31 ± 0.02 ^g^
Trolox	4.25 ± 0.08 ^a^	7.32 ± 0.26 ^a^	44.41 ± 0.11 ^a^

^a–l^ Different superscript letters within each column indicate significant differences (Tukey’s test, *n* = 3, *p* < 0.05). C: cob extract; H: husk extract; S: silk extract; CT: butterfly pea extract; CT + C: butterfly pea and cob extract; CT + H: butterfly pea and husk extract; CT + S: butterfly pea and silk extract; C + H: cob and husk extract; C + S: cob and silk extract; H + S: husk and silk extract; C + H+ S: cob, husk, and silk extract; mixed: combination of butterfly pea, cob, husk, and silk extracts.

**Table 4:** Pearson’s correlation coefficient (*r*) values of phytochemical compositions and antioxidant capacities (ABTS, DPPH and FRAP) of different extracts obtained from purple corn and butterfly pea.

Phytochemical Compositions	Pearson’s Correlation Coefficient (*r*)		
	ABTS	DPPH	FRAP
Total phenolic content (TPC)	0.739 *	0.960 *	0.823 *
Total anthocyanin content (TAC)	0.098	0.170	0.613 *
Delphinidin (Del)	0.229	0.334	0.627 *
Tryptophan (Trp)	0.113	−0.005	0.331
Serotonin (5-HT)	−0.016	−0.047	0.413
Melatonin (MLT)	−0.150	−0.088	0.438
Gallic acid (GA)	0.033	0.191	−0.078
Protocatechuic acid (PCCA)	0.663 *	0.942 *	0.794 *
*p*-Hydroxybenzoic acid (*p*-HO)	0.519	0.686 *	0.545
Chlorogenic acid (ChA)	0.039	−0.014	0.244
Vanillic acid (VA)	0.370	0.457	0.560
*p*-Coumaric acid (*p*-CA)	−0.092	0.057	0.199
Ferulic acid (FA)	−0.191	0.014	0.154
Sinapic acid (SA)	−0.492	−0.703 *	−0.699 *

* Corresponds to significance at *p* < 0.05.

**Table 5:** Composition of each extraction sample and its extraction yield.

Sample Code	Dried Material (g)				Water (mL)
	Butterfly Pea (CT)	Silk (S)	Husk (H)	Cob (C)	
C	-	-	-	6.50	390
H	-	-	6.50	-	390
S	-	6.50	-	-	390
CT	6.50	-	-	-	390
CT + C	3.25	-	-	3.25	390
CT + H	3.25	-	3.25	-	390
CT + S	3.25	3.25	-	-	390
C + H	-	-	3.25	3.25	390
C + S	-	3.25	-	3.25	390
H + S	-	3.25	3.25	-	390
C + H + S	-	2.17	2.17	2.17	390
Mixed *	1.65	1.65	1.65	1.65	390

* Mixed: combination of butterfly pea, cob, husk, and silk extracts.

## Data Availability

Not applicable.
